# Amelioration of Hypercholesterolemia by an EGFR Tyrosine Kinase Inhibitor in Mice with Liver-Specific Knockout of *Mig-6*

**DOI:** 10.1371/journal.pone.0114782

**Published:** 2014-12-08

**Authors:** Jun Choul Lee, Byung Kil Park, Sorim Choung, Ji Min Kim, Kyong Hye Joung, Ju Hee Lee, Koon Soon Kim, Hyun Jin Kim, Jae-Wook Jeong, Sang Dal Rhee, Bon Jeong Ku

**Affiliations:** 1 Department of Internal Medicine, Chungnam National University School of Medicine, Daejeon, Korea; 2 Department of Internal Medicine, Daejeon Veterans Hospital, Daejeon, Korea; 3 Department of Drug Development and Discovery, Graduate School of New Drug Development and Discovery, Chungnam National University, Daejeon, Korea; 4 Department of Obstetrics, Gynecology and Reproductive Biology, Michigan State University, Grand Rapids, Michigan, United States of America; 5 Research Center for Drug Discovery Technology, Division of Drug Discovery Research, Korea Research Institute of Chemical Technology, Daejeon, Korea; Baylor College of Medicine, United States of America

## Abstract

Mitogen-inducible gene 6 (*Mig-6*) is a negative feedback inhibitor of epidermal growth factor receptor (EGFR) signaling. We previously found that *Mig-6* plays a critical role in the regulation of cholesterol homeostasis and in bile acid synthesis. In this study, we investigated the effects of EGFR inhibition to identify a potential new treatment target for hypercholesterolemia. We used a mouse model with conditional ablation of the *Mig-6* gene in the liver (Alb*^cre/+^Mig-6^f/f^*; *Mig-6^d/d^*) to effectively investigate the role of *Mig-6* in the regulation of liver function. *Mig-6^d/d^* mice were treated with either the EGFR inhibitor gefitinib or statin for 6 weeks after administration of a high-fat or standard diet. We then compared lipid profiles and other parameters among each group of mice. After a high-fat diet, *Mig-6^d/d^* mice showed elevated serum levels of total cholesterol, high-density lipoprotein (HDL) cholesterol, low-density lipoprotein (LDL) cholesterol, triglycerides and glucose, characteristics resembling hypercholesterolemia in diabetic patients. We observed decreases in serum levels of lipids and glucose in high-fat-diet-fed *Mig-6^d/d^* mice after 6 weeks of treatment with gefitinib or statin. Furthermore gefitinib-treated mice showed significantly greater decreases in serum levels of total, HDL and LDL cholesterol compared with statin-treated mice. Taken together, these results suggest that EGFR inhibition is effective for the treatment of hypercholesterolemia in high-fat-diet-fed *Mig-6^d/d^* mice, and our findings provide new insights into the development of possible treatment targets for hypercholesterolemia via modulation of EGFR inhibition.

## Introduction

Hypercholesterolemia and dyslipidemia are common risk factors for cardiovascular disease, which is a leading cause of illness and death worldwide. In the majority of people with hypercholesterolemia among the general public, the condition is attributable to a high-fat diet and to poorly understood susceptibility and modifier genes. Defining the molecular mechanisms regulating cholesterol homeostasis will lead to more effective methods of treating and preventing cardiovascular disease [1]. Statins have been the drugs of choice for decreasing plasma cholesterol levels, leading to substantial improvements in cardiovascular morbidity and mortality. However, certain patients are unable to tolerate statins, such as those with refractory familial hyperlipidemia, who are intolerant to all statin therapies [2].

Epidermal growth factor receptor (EGFR) signaling controls morphogenesis and/or homeostasis processes, including survival, proliferation, migration, and differentiation, in several tissues. Because of the capacity of EGFR signaling to promote numerous critical biological outcomes, dysregulation of this pathway has been implicated in many human diseases [3], [4]. Mitogen-inducible gene 6 (*Mig-6*) is an immediate early response gene encoding a non-kinase scaffolding adaptor protein induced by various mitogens, stressors and hormones that acts as a negative feedback inhibitor of EGFR signaling through its direct, physical interaction with EGFR [5]–[7].

Previously, we found that mice with conditional ablation of *Mig-6* in the liver have abnormalities related to cholesterol metabolism, such as hyperlipidemia, characterized by marked increases in LDL cholesterol, intrahepatic lipids and hepatomegaly [8]. However, the roles of EGFR and EGFR kinase inhibitors in hypercholesterolemia have not been studied systematically. The goals of this study were to determine the effects of an EGFR tyrosine kinase inhibitor, compared with statin, in a *Mig-6^d/d^* hypercholesterolemia mouse model fed a high-fat diet. In this study, we found that the EGFR tyrosine kinase inhibitor gefitinib improved hypercholesterolemia and insulin resistance in high-fat-diet-fed *Mig-6^d/d^* mice. These results indicate a novel relationship between EGFR and hypercholesterolemia and suggest a new hypolipidemic drug with a mechanism of action differing from that of statin.

## Materials and Methods

### Ethics statement

All animal research was conducted according to protocols approved by Chungnam National University Hospital's Institutional Animal Care and Use Committees, and the Guideline for the Care and Use of Laboratory Animal was observed. The Chungnam National University Hospital's Institutional Animal Care and Use Committees specifically approved this study (Permit Number: CNUH-A0014).

### Animals and Tissue collection

*Mig-6* “floxed” (Mig-6*^f/f^*) mice and *Alb^cre/+^Mig-6^f/f^ (Mig-6^d/d^)* mice [9] were maintained in the designated animal care facility at the Chungnam National University School of Medicine according to the institutional guidelines for the care and use of laboratory animals. The mice were maintained with consistent temperature (23°C) on a 12-h light/12-h dark cycle (0600 h/1800 h). All mice received standard chow before the initiation of the experimental procedures. The *Mig-6^d/d^* male mice were randomly divided in two groups. The normal control group (N-C) was maintained with standard rodent chow, and the other group received a high-fat diet, in which 60% of the Kcal came from fat (D12492, Research Diet, USA). After 16 weeks receiving standard diet or high-fat diet, high-fat diet mice were randomly divided into control (Con), gefitinib plus high-fat diet (G) treatment (10 mg/kg/day) or statin plus high-fat diet (S) treatment (simvastatin, 20 mg/kg/day) group. At the ends of 6^th^ week, all mice of N-C, Con, G and S were sacrificed. The serums were collected to examine the serum biochemical markers, and liver and fat specimens were weighed at the time of sacrifice. Livers were obtained to observe hepatic pathological changes.

### Tissue Staining

For Hematoxylin/Eosin (H&E) Staining, livers were fixed overnight in 4% paraformaldehyde, followed by thorough washing in 70% ethanol. The tissues were processed, embedded in paraffin, and sectioned. Five micrometer sections were cut and stained with hematoxylin and eosin by standard protocols.

### Western Blot Analysis

Mouse liver tissues were washed with PBS solution and homogenized in a buffer containing 10 mM Tris-HCl (pH 7.4), 150 mM NaCl, 2.5 mM EDTA, and 0.125% Nonidet P-40 (vol/vol). Cellular debris was removed by centrifugation at 14,000 rpm for 15 min at 4 uC. Protein concentration was determined by Bradford's method using BSA as the standard. Samples containing 30 mg proteins were applied to 10% SDS-PAGE. The separated proteins were then transferred onto a polyvinylidene difluoride membrane (Millipore Corp., Bedford, MA). Membranes were blocked overnight with 5% skim milk (wt/vol) in PBS with 0.1% Tween 20 (vol/vol) (Sigma-Aldrich, St. Louis, MO) and probed with rabbit-anti-Mig-6 (Sigma-Aldrich, St. Louis, MO), EGFR (Cell signaling, Danvers, MA), and AKT (Cell signaling, Danvers, MA). Immunoreactivity was visualized by incubation with a horse-radish peroxidase-linked second antibody and treatment with ECL reagents. To control for loading, the membrane was stripped, probed with mouse-anti-ß-actin (Sigma-Aldrich, St. Louis, MO) and developed again.

### Serum chemistry

Serum was collected from the orbital sinus using disposable Pasteur pipets (Fisher Scientific, Pittsburgh, PA) after a 24 hour fasting period, placed in serum collecting tubes (BD, Franklin Lakes, NJ), centrifuged at 1,200× g for 10 min at 4°C, and stored at −20°C prior to analyze serum biochemical markers.

### Statistical analysis

The results are expressed as means ±SEM. Statistical analysis was performed using SPSS ver. 18.0 (SPSS Inc., Chicago, IL, US). The Mann-Whitney *U*-test was used to compare differences between the two groups. A *P*-value of <0.05 was considered to indicate statistical significance.

## Results

### Generation of conditional ablation of Mig-6 in the liver using *Alb^cre^* and impact of gefitinib on adipose tissue and liver enzymes in *Mig-6^d/d^* mice

**Figure 1:**
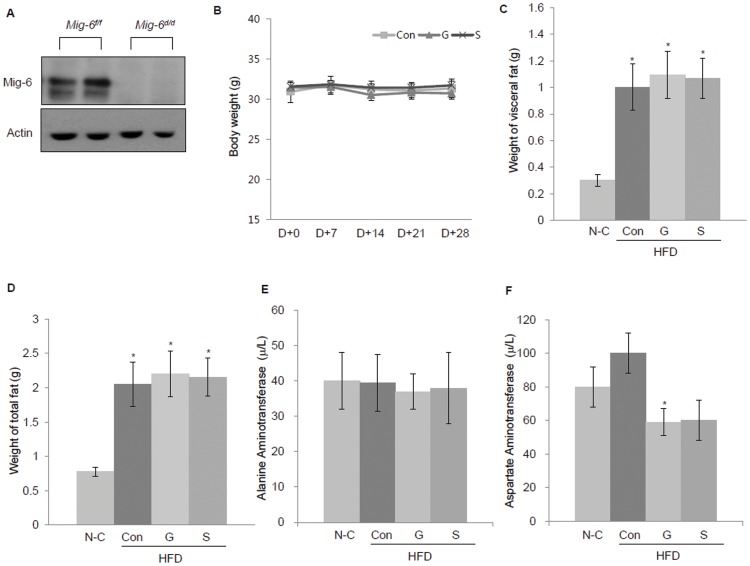
Generation of conditional ablation of Mig-6 in the liver and impact of gefitinib on adipose tissue and liver enzymes in *Mig-6^d/d^* mice. (A) Western blot analysis of Mig-6 in the liver of Mig-6^f/f^ and Mig-6^d/d^ mice. Liver tissue from Mig-6^f/f^ and Mig-6^d/d^ mice were lysed and equal amounts of protein were subjected to SDS-PAGE and Western blot analysis for Mig-6. (B) Changes of body weight during the course of standard diet or high fat-diet with or without gefitinib or statin. (C) Changes in visceral fat and (D) total fat weight after 6 weeks of gefitinib and statin treatment. (E) Relative changes in plasma alanine aminotransferase and (F) aspartate aminotransferase after 6 weeks of gefitinib and statin treatment. Values represent the means ±SEM. *P<0.05 relative to the normal control. N-C, normal control; HFD, high-fat diet; Con, control; G, gefitinib; S, statin.

Previously, to investigate the role of *Mig-6* in the regulation of liver function and metabolism [8], we generated conditional ablation of *Mig-6* (Alb*^cre/+^Mig-6^d/d^*) in the liver of mice (Fig. 1A). *Mig-6^d/d^* mice received a standard diet (N-C) or high-fat diet for 16 weeks. The high-fat-diet-fed mice were then randomly divided into the following three groups and treated for an additional 6 weeks: high-fat diet only (control; Con), gefitinib plus high-fat diet (G) and statin plus high-fat diet (S). Neither gefitinib nor statin had any effect on body weight or food intake among the high-fat diet mice (Fig. 1B).

The high-fat-diet-fed *Mig-6^d/d^* mice showed increases visceral fat and total fat weights compared with the standard-diet-fed *Mig-6^d/d^* mice, and gefitinib or statin treatment for 6 weeks failed to decrease these weights (Fig. 1C, D). We also examined the level of liver enzymes after gefitinib or statin treatment to evaluate the side effect of the both drugs on *Mig-6^d/d^* mice. There were no increases in alanine aminotransferase (ALT) or aspartate aminotransferase (AST) after the gefitinib or statin treatment showing there is no side effects both drugs during the experiment (Fig. E, F).

### The effects of an EGFR tyrosine kinase inhibitor gefitinib on glucose tolerance and insulin action

**Figure 2:**
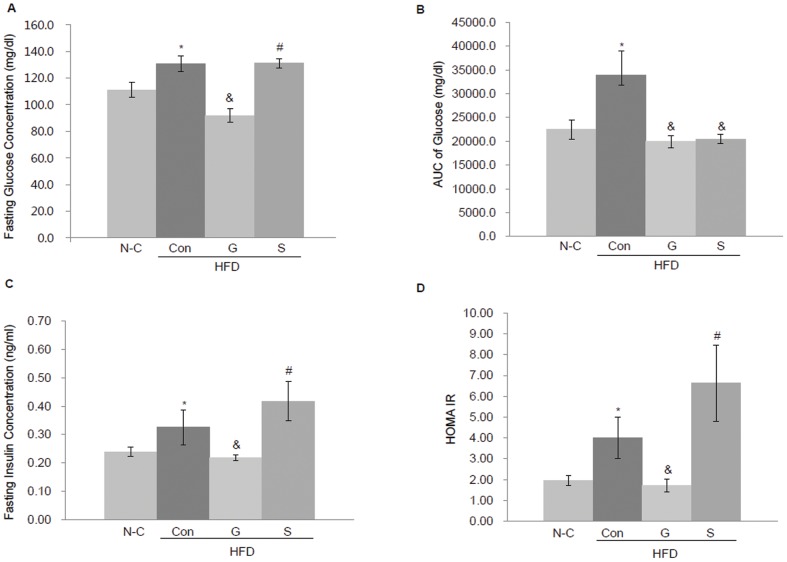
Glucose and insulin tolerance analyses. Changes in glucose and insulin concentrations after 6 weeks of gefitinib or statin treatment. (A) Fasting glucose concentration. (B) AUC for glucose levels following oral glucose tolerance test. (C) Fasting insulin concentration. (D) The insulin resistance index HOMA-IR. n = 10 each. Values represent the means ±SEM. *P<0.05 relative to N-C, ^&^ *P*<0.05 relative to Con (high-fat diet only group), ^#^ *P*<0.05 relative to G (gefitinib plus high-fat diet group). N-C, normal control; HFD, high-fat diet; Con, control; G, gefitinib; S, statin.

After 6 weeks treatment, metabolic analyses showed that control (high-fat diet only) *Mig-6^d/d^* mice had an increased fasting glucose (Fig. 2A) and glucose area under the curve (AUC) following an oral glucose tolerance test (Fig. 2B). Also the mice showed an increased fasting insulin concentration (Fig. 2C) and HOMA-IR index, a measure of insulin resistance (Fig. 2D) compared with the normal control (standard diet) mice. Gefitinib treatment, even when combined with a high-fat diet, reduced the fasting insulin concentration, HOMA IR index and glucose intolerance to the levels seen in the standard diet group, indicating that gefitinib potentially improves insulin resistance in diabetic condition with dysregulated EGFR signaling. The statin plus high-fat diet *Mig-6^d/d^* mice also showed a reduction in the glucose intolerance, but not in fasting insulin concentration or the HOMA IR index, compared with gefitinib-treated mice (Fig. 2C,D).

In wild type high-fat diet-fed mice, gefitinib reduced the fasting level of serum insulin and glucose. Also, we investigated whether gefitinib could improve lipid profiles in wild type mice. Compared with the control mice, there was no increase of serum total cholesterol and triglyceride in gefitinib-treated wild type mice even after 6 weeks of a high-fat diet (S1 Figure).

### Effects of gefitinib on liver weight and histology in *Mig-6^d/d^* mice

**Figure 3:**
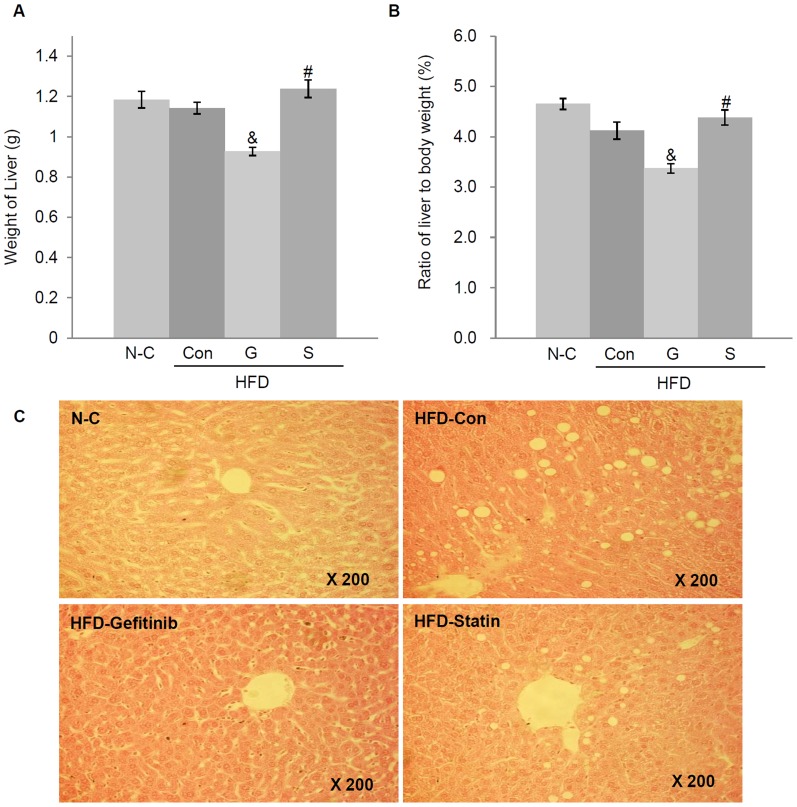
Effects of gefitinib and statin treatments on liver pathology. Changes in the weight and pathology of the liver after 6 weeks of gefitinib or statin treatment. (A) Weight of the liver. (B) Weight of the liver after body weight adjustment. (C) Hematoxylin-eosin staining. All photomicrographs are ×200 magnification. Values represent the means ±SEM. ^&^ *P*<0.05 relative to Con, ^#^ *P*<0.05 relative to G. N-C, normal control; HFD, high-fat diet; Con, control; G, gefitinib; S, statin.

We assessed the impact of gefitinib or statin on the liver tissues of high-fat-diet-fed *Mig-6^d/d^* mice. The liver weights of *Mig-6^d/d^* mice after gefitinib treatment plus a high-fat diet were significantly decreased compared with the high-fat diet only or statin plus high-fat-diet-fed *Mig-6^d/d^* mice (Fig. 3A). In addition, body-weight-adjusted liver weights were decreased in the gefitinib plus high-fat diet *Mig-6^d/d^* mice (Fig. 3B).

To further analyze liver phenotypes among the groups, the liver histology was examined. In hematoxylin-eosin staining of liver tissues, many vacuolated lesions were found near the central veins of the high-fat-diet-fed *Mig-6^d/d^* mouse livers, in resemblance to fatty livers, compared with the standard-diet-fed *Mig-6^d/d^* mice. However, the gefitinib plus high-fat-diet-fed *Mig-6^d/d^* mice showed a notable decrease in macrovesicular steatosis of the liver compared with the high-fat diet only and statin plus high-fat-diet-fed *Mig-6^d/d^* mice (Fig. 3C).

### Changes in serum lipid profiles after gefitinib treatment in *Mig-6^d/d^* mice

**Figure 4:**
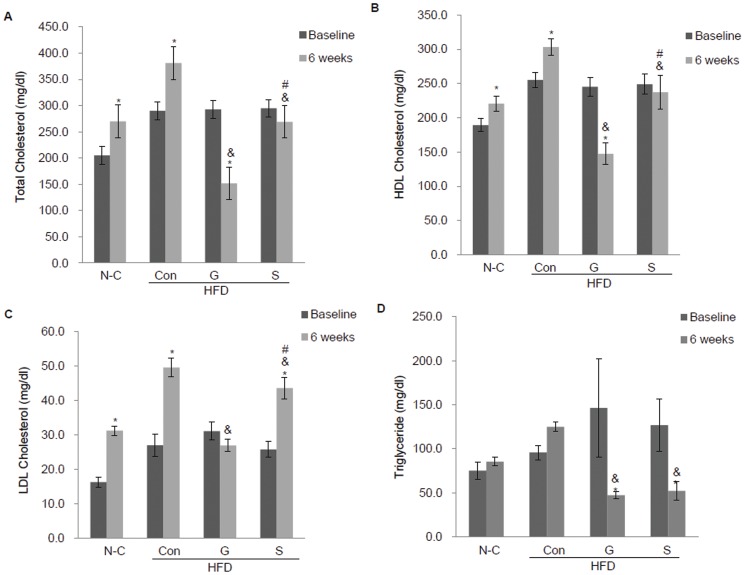
Effects of gefitinib and statin treatments on the serum lipid profile. Changes in serum lipids after 6 weeks of gefitinib or statin treatment. (A) Total cholesterol, (B) HDL cholesterol, (C) LDL cholesterol, (D) triglyceride levels (n = 10 each, *P*<0.05). Values represent the means ±SEM. *P<0.05 relative to baseline. ^&^ *P*<0.05 relative to Con, ^#^ *P*<0.05 relative to G. N-C, normal control; HFD, high-fat diet; Con, control; G, gefitinib; S, statin.

To assess the effects of gefitinib on cholesterol metabolism in *Mig-6^d/d^* mice, we examined the profiles of several lipids, including total cholesterol, HDL cholesterol, LDL cholesterol and triglycerides, after feeding of a standard- or high-fat diet combined with statin or gefitinib. At week 16, high-fat-diet-fed *Mig-6^d/d^* mice showed significantly higher levels of serum total, HDL, and LDL cholesterol and triglycerides, compared with the standard-diet-fed *Mig-6^d/d^* mice. We then investigated whether gefitinib could improve lipid profiles in *Mig-6^d/d^* mice. Compared with statin, gefitinib notably decreased serum total, HDL and LDL cholesterol levels after 6 weeks of treatment in *Mig-6^d/d^* mice fed a high-fat diet (Fig. 4A, B, C). Gefitinib also decreased triglyceride levels, but there was no significant difference compared with statin-treated *Mig-6^d/d^* mice (Fig. 4D). The serum chemistry profiles suggest that EGFR signaling is responsible for lipid metabolism, and EGFR tyrosine kinase inhibitors may improve the hypercholesterolemia induced by dysregulated EGFR signaling.

### Changes in EGFR activities after gefitinib treatment in *Mig-6^d/d^* mice

**Figure 5:**
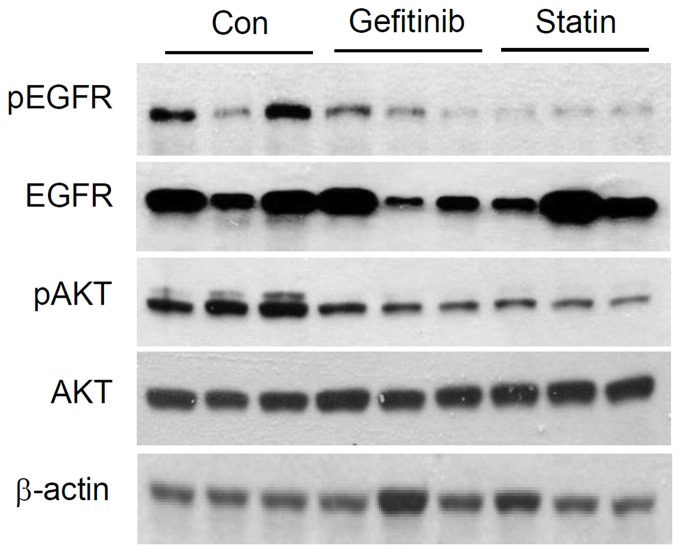
Regulation of EGFR expression and Akt signaling by gefitinib. Western blotting analysis of EGFR, pEGFR, Akt and pAkt levels after gifitinib or statin treatment. ß-actin is included as a lading control.

To confirm the effect in molecular level, we checked changes of EGFR and AKT protein levels after gefitinib or statin treatment. As we expected, phosphorylation levels of EGFR and AKT were decreased in Mig-6*^d/d^* mice after statin or gefitinib treatment (Fig. 5).

## Discussion

In this study, we demonstrated that use of the EGFR tyrosine kinase inhibitor, gefitinib, in high-fat-diet-fed *Mig-6^d/d^* mice for 6 weeks induced a marked improvement in hypercholesterolemia and insulin resistance accompanied by improved intrahepatic lipid levels. This result provides the possibility that inhibiting EGFR tyrosine signaling can be another promising treatment modality of the future for hypercholesterolemia, type 2 diabetes mellitus, or fatty liver.

Hypercholesterolemia is a common risk factor for cardiovascular disease. Therefore, the treatment of hypercholesterolemia should focus not only on controlling the numerical factors, such as serum cholesterol levels but also on lowering the overall cardiovascular risk [10]–[12]. Hypercholesterolemia usually results from nutritional factors, such as obesity and a diet high in saturated fats, combined with an underlying polygenic predisposition, but sometimes it can stem entirely from genetic causes, such as the case with monogenic familial hypercholesterolemia [11]. Currently, statins are the drugs of choice to decrease serum cholesterol levels and reduce the risk of cardiovascular disease and death. Unfortunately, statin therapy has several limitations. First, statins can cause insulin resistance and increase the risk of type 2 diabetes mellitus. Second, certain populations do not respond to statin treatment due to differences in genotypes and epigenetics [12]. Third, statins may induce myopathy, elevation of liver enzymes. Finally, even the reduction of cholesterol by statins does not improve fatty liver. Therefore, a new approach to hypercholesterolemia treatment is needed.

Signaling through EGFR is properly regulated and precisely coordinated by the various ligands and negative feedback regulators of EGFR, because excessive or deficient signaling can result in some of the most severe diseases. The EGFR pathway is crucial in normal growth of human organs but under certain conditions, EGFR serves as a stimulus for cancer growth [13]–[15]. Therefore the EGFR pathway has been widely studied and variety of anti-EGFR agents was developed. But still, the research on developing therapies and drugs involving EGFR and EGFR signaling are currently under investigation [16]–[19]. *Mig-6* is a non-kinase scaffolding adaptor protein found in the cytosol that acts as a negative feedback inhibitor of EGFR signaling through its direct, physical interaction with this receptor [20]–[23]. Recent discoveries showed roles for *Mig-6* in stress responses, tissue homeostasis, and cancer development, indicating that it may be critical for the regulation of many cellular responses. However, its biological and pathophysiological roles in human diseases need to be elucidated [24], [25].

In this study, we found that *Mig-6* ablation in the liver induces a fatty liver phenotype and disruption of cholesterol homeostasis by upregulation of EGFR signaling pathway after a high-fat diet, implicating a relationship between the EGFR signaling pathway and cholesterol metabolism. And, treatment with the EGFR tyrosine kinase inhibitor gefitinib significantly decreased total, HDL, and LDL cholesterol and triglyceride levels in *Mig-6^d/d^* mice more effectively than did statin. Although there were no significant changes in visceral, subcutaneous and total adipose weights with gefitinib treatment, there were significant decreases in intrahepatic lipid deposits and liver weight.

Moreover, gefitinib treatment in high-fat diet *Mig-6^d/d^* mice showed decreases in fasting insulin concentration and insulin resistance, suggesting gefitinib may improve metabolic syndrome in those with dysregulated EGFR and/or its signaling pathway. This study also provides the evidence for the use of EGFR tyrosine kinase inhibitors in hypercholesterolemia patients who do not fully controlled or resistant to conventional statin treatment.

A recent study demonstrated the efficacy of EGFR tyrosine kinase inhibitors, as well as the associated molecular mechanisms, on diabetes control and insulin action in high-fat-diet-fed mice, and suggested that EGFR and/or its signaling pathway may have a role in insulin resistance in obesity and diabetes; those results support our own regarding the possible role of EGFR tyrosine kinase inhibitors in metabolic disorders [26]. Consistent with the previous study [27], we demonstrated both gefitinib and statin inhibited both EGFR and AKT activation. This result suggests the possibility that statin inhibits the synthesis of cholesterol in liver and also lowers the serum cholesterol by inhibiting EGFR and AKT signaling pathway. So far, there is no medication that can treat both hypercholesterolemia and fatty liver which are often accompanied in patients with diabetes. For example, metformin has recently received increased attention because of its potential antitumorigenic effects on several cancers by inactivation of mTOR and suppression of its downstream effectors. Similarly, gefitinib, although first developed as anticancer agent, this study provides a new insights into the understanding the pathophysiology of cholesterol and fat metabolism in diabetes and a possible novel target in treating hypercholesterolemia and fatty liver in diabetes patients.

There have been no previous reports on the relationship between EGFR tyrosine kinase inhibitor and hypercholesterolemia pathophysiology other than this study. However our study has limitations that we did not access the detailed molecular mechanism of EFGR tyrosine inhibitor on improvement of hypercholesterolemia and more *in vivo* studies are need to elucidate that EGFR tyrosine kinase improves hypercholesterolemia in patients.

In summary, our study showed that ablation of *Mig-6* in the liver results in multiple metabolic phenotypes, and that treatment with an EGFR tyrosine kinase inhibitor improved hypercholesterolemia and insulin resistance, suggesting a possible role for EGFR signaling in cholesterol metabolism. By this study, we demonstrated a novel relationship between EGFR and hypercholesterolemia and provided new insight into possible treatment targets for hypercholesterolemia via modulation of EGFR inhibition, the mechanism of action of which differs from that of statins. Finally, further studies are needed to investigate EGFR signaling dysregulation in patients with hypercholesterolemia who do not respond to statins.

## Supporting Information

S1 Figure **Effects of gefitinib on serum glucose and lipid in wild type mice.** Changes in glucose, insulin concentrations and serum lipids after 6 weeks of gefitinib treatment. (A) Fasting glucose concentration. (B) Fasting insulin concentration. (C) Triglyceride levels. (D) Total cholesterol. n = 10 each. Values represent the means ±SEM. *P<0.05 relative to control.
(TIF)
